# Pharmacophore study, molecular docking and molecular dynamic simulation of virgin coconut oil derivatives as anti-inflammatory agent against COX-2

**DOI:** 10.1186/s40538-022-00340-0

**Published:** 2022-10-18

**Authors:** Kho Swen Jack, Mohd Razip Bin Asaruddin, Showkat Ahmad Bhawani

**Affiliations:** Faculty of Resource Science and Technology, Universiti Malaysia Sarawak, 94300 Kota Samarahan, Sarawak Malaysia

**Keywords:** Virgin coconut oil, COX-2, inflammation, Pharmacophore, Monolaurin, Molecular docking, Molecular dynamics simulation

## Abstract

**Background:**

Virgin coconut oil is mostly made up of saturated fatty acids in which approximately 72% are medium chain triglycerides. Medium chain triglycerides can be digested into medium chain fatty acids and medium chain monoglycerides which are bioactive components. Therefore, it is very important to study the in-silico ability of some Virgin coconut oil derivatives, namely, medium chain fatty acids and medium chain monoglycerides to inhibit Cyclooxygenase 2 (COX-2) protein for prevention of excessive inflammatory response.

**Results:**

Pharmacophore study displayed monolaurin with two hydrogen bond donor, three hydrogen bond acceptor and five hydrophobic interactions, while lauric acid presented two hydrogen bond acceptor, five hydrophobic interactions and a negative ion interaction. Molecular docking underlined the ability of monolaurin in the inhibition of COX-2 protein which causes inflammatory action with a decent result of energy binding affinity of − 7.58 kcal/mol and 15 interactions out of which 3 are strong hydrogen bond with TYR385 (3.00 Å), PHE529 (2.77 Å), and GLY533 (3.10 Å) residues of the protein. Monolaurin was employed as hydrogen bond acceptor to the side of residue TYR385 of COX-2 protein with an occupancy of 67.03% and was observed to be long-living during the entire 1000 frames of the molecular dynamic simulation. The analysis of RMSD score of the Monolaurin–COX-2 complex backbone was calculated to be low (1.137 $\pm$ 0.153 Å) and was in a stable range of 0.480 to 1.520 Å. Redocking of this complex still maintained a strong hydrogen bond (2.87 Å) with the main residue TYR385. AMDET results where promising for medium chain fatty acids and medium chain monoglycerides with good physicochemical drug scores.

**Conclusions:**

This can be concluded from the results obtained that the monolaurin has strong interactions with COX-2 protein to disrupt its function due to significant hydrogen bonds and hydrophobic interactions with amino acid residues present in the target protein’s active site. These results displayed a very significant anti-inflammatory potential of monolaurin and a new promising drug candidates as anti-inflammatory agent.

**Graphical Abstract:**

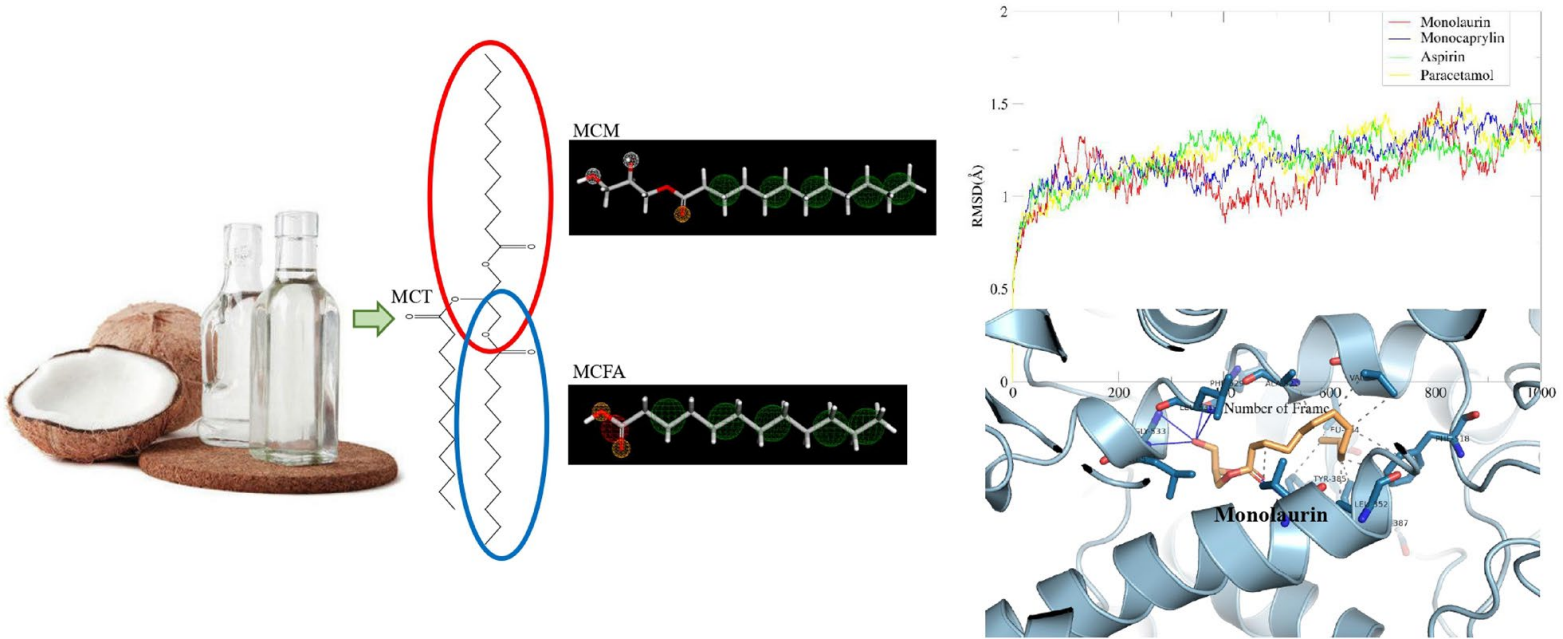

## Background

In more than 80 countries in the tropics, coconut (*Cocos nucifera*) is one of the most important oil-producing trees. As a multipurpose tree, it is well-liked by the general public. Virgin coconut oil (VCO) is the oil of ripe coconut in its purest form, and it possesses plenteous substantial health advantages particularly due to the high concentration of medium chain triglycerides (MCTs) which are derivable to medium chain fatty acids (MCFAs) and medium chain monoglycerides (MCMs). Dicapricmonolaurin (14.32%), dilauricmonocaprin (18.59%), trilaurin (21.88%), dilauricmonomyristin (17.20%), dimyristicmonolaurin (9.62%) were reported as the main triglyceride components of VCO [1]. The MCFAs of VCO includes caproic acid, caprylic acid, capric acid, lauric acid and myristic acid. MCFAs molecules have great digestibility in the body due to the complete hydrolysis of MCTs into glycerol, monoglycerides, and fatty acids by pancreatic enzyme, namely, lipase [2]. MCMs are compounds having a single carbon chain length ranging from 8 to 14 atoms in the acyl groups, such as monocaprylin, monolaurin, monocaprin, and monomyristin [3]. MCMs and MCFAs are bioactive forms of MCTs with versatile diverse pharmacological propensities that can help in boosting the immune system, treating various disorders such as inflammatory, gastrointestinal, cardiovascular and have the capability to fight off numerous bacterial, fungal and viral infections [3]. Therefore, research, health, and food supplements are starting to rely heavily on VCO-derived MCFAs and MCMs [4].

The natural response to infection or tissue injury is known as inflammation. It is normally restricted to the damaged tissues, but it can become uncontrolled in disorders such as asthma, nephritis, type-2 diabetes mellitus, malignancies and Alzheimer's disease if not dealt with appropriately [5]. Analgesics, such as narcotics and opioids, and nonsteroidal anti-inflammatory medications (NSAIDs), otherwise Ibuprofen and naproxen, are classically used to treat inflammation. Lot of focus on COX-2 inhibitors in the treatment of inflammation has been taken into consideration. Arachidonic acid is converted into prostaglandins by COX-2, which is well-known for its role in cascading inflammation [6]. It has also been proven in various studies that COX inhibitors are potentially cancer preventative particularly when they are selective against COX-2 with the pharmacological anti-inflammatory effect of NSAIDs mediated by the COX-2 inhibition [7]. Inflammation has been treated for decades with a variety of COX inhibitors, namely, a cetylsalicylic acid and indomethacin. However, some side effects, including as bronchospasm, angioedema and renal dysfunction, as well as bone marrow depression, headache and vertigo, have been reported during their use [8, 9]. As a result, finding novel and innovative COX-2 inhibitors with enhanced potency and selectivity is a source of growing worry for the medical community.

There has only been a short list of reported studies on the anti-inflammatory properties of VCO. In vivo studies regarding severe inflammation based on rat models given VCO were demonstrated by researchers with promising results especially with fermented VCO [10–13]. In vitro studies of inflammation using VCO as suppresser were also reported [14, 15]. To search and create COX-based anti-inflammatory and anticancer medicines, several CADD or Computer Aided Drug Design methods have always been utilized. Virtual screening and docking-based technique were performed to find suitable COX-2 inhibitor [5, 16]. On the other hand, Schaller et al. (2020) used pharmacophore modelling combining ligand and structure-based models to screen out potential COX-2 inhibitor [5, 17]. A few studies also reported in silico COX-2 inhibitory effect using the protein 5F1A included with molecular docking and molecular dynamics simulation [7, 18–21]. In silico studies and in vitro studies were performed by Murugesan et al. [18], to search for medication for rheumatoid arthritis (severe inflammation) that is more effective, while also having less side effects and toxicities.

As a result of the previously mentioned problem, a computational drug design was employed in this study to find possible VCO derivative as COX-2 inhibitors. The ligand-based pharmacophore presentation was used to exhibit pharmacophores of MCMs, MCFAs and some reference drug. To establish the exact interaction of MCMs and MCFAs, the molecular docking protocol was employed to explore the binding affinity, hydrogen bond and orientation of VCO small molecules with COX-2 protein. Molecular dynamic simulation (MDS) was employed to determine the stability of the complex formed by the best binding small molecules with COX-2 protein followed by a post redocking. Finally, ADME was computed to determine the drug likeliness and toxicity of the test compounds.

## Materials and methods

### Pharmacophore study

The 3D structures of VCO derivatives such as MCFAs (caproic acid, capric acid, caprylic acid, lauric acid, and myristic acid), MCMs (monolaurin, monocaprin, monocaproin, monocaprylin, and monomyristin), and reference drugs (aspirin, paracetamol, and ibuprofen) were downloaded via PubChem and saved as sdf files. The protein model of COX-2 protein (5F1A) and ligands files were loaded into Pharmit (https://pharmit.csb.pitt.edu/index.php) for receptor and features pharmacophore visualization.

### Preparation of receptors for molecular docking

**Fig. 1:**
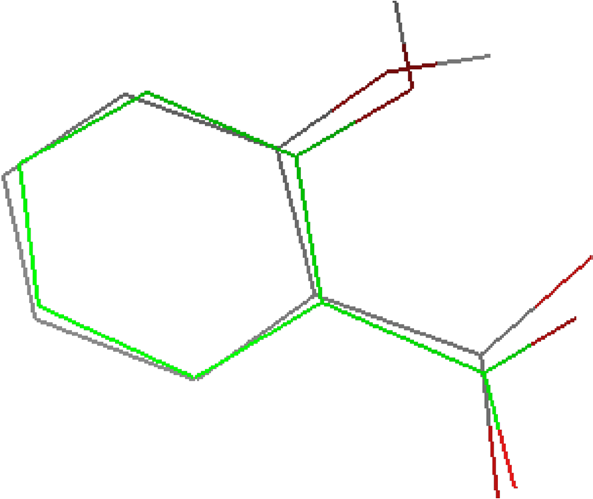
Comparison of the superimposed native ligand (grey) and redock ligand (green) of 5F1A

The 3D model of COX-2 protein (5F1A) was download via Protein Data Bank (PDB) and saved as pdb file type. The protein has a resolution of 2.38 Å and built with two chains, namely, chain A and B. Validation was determined by the best redocked pose’s RMSD of the original ligand which should be less than 2.0 Å from the experimental data so the protein can be valid for further experimentation [23]. Docking of the native co-crystallized ligands was, therefore, explored to validate the docking accuracy by examining how closely the best docked orientation superimpose the original ligand in a biological way. Hegazy et al. (2012) stated that a smaller RMSD values was obtained from flexible docking with AutoDock 4.2 which is more accurate and thus more similar to biological co-crystallization [24]. The redocking for protein 5F1A was successful. In Fig. 1, the salicylic acid co-crystallized with COX-2 appears precisely overlaid on the natively attached versions. The salicylic acid docked into COX-2 obtained a RMSD of 0.929 Å using a flexible docking strategy which was lower than 2.0 Å and valid to be used for docking. As a control, redocking was also done with the protein 5IKV obtaining a RMSD value of 1.682 Å. In both cases, the RMSD values are significantly smaller than the 2.38 Å and 2.51 Å that represent the X-ray resolution of the relevant protein, namely, 5f1A and 5IKV, respectively. These numbers support the validity of this technique and showed that the developed model correlates well with the experimental X-ray crystal structures [20].

### Preparation of ligands for molecular docking

The 3D structures of VCO derivatives such as MCFAs (caproic acid, capric acid, caprylic acid, lauric acid, and myristic acid), MCMs (monolaurin, monocaprin, monocaproin, monocaprylin, and monomyristin), and reference drugs (aspirin, paracetamol, and ibuprofen) were downloaded via PubChem and saved as sdf files. Then, the software Open Babel was utilized to change all ligand files into pdb file type and followed using Aurodock to convert the files into pdbqt file type.

AutoDock Vina 4.2 was employed to conduct molecular docking analysis of the selected ligand molecule with the protein targets. When the COX-2 protein receptor was first inserted in the software, the receptor was prepared via a series of processes for dockings in the following orders, removal of all water, inhibitors, and followed by polar hydrogen bond (HB) optimization. After inserting the polar HB, kollman charges were added. The file of the prepared protein receptor was saved as pdbqt file type and named after the protein, such as 5f19.pdbqt (COX-2).

### Active site determination

**Fig. 2:**
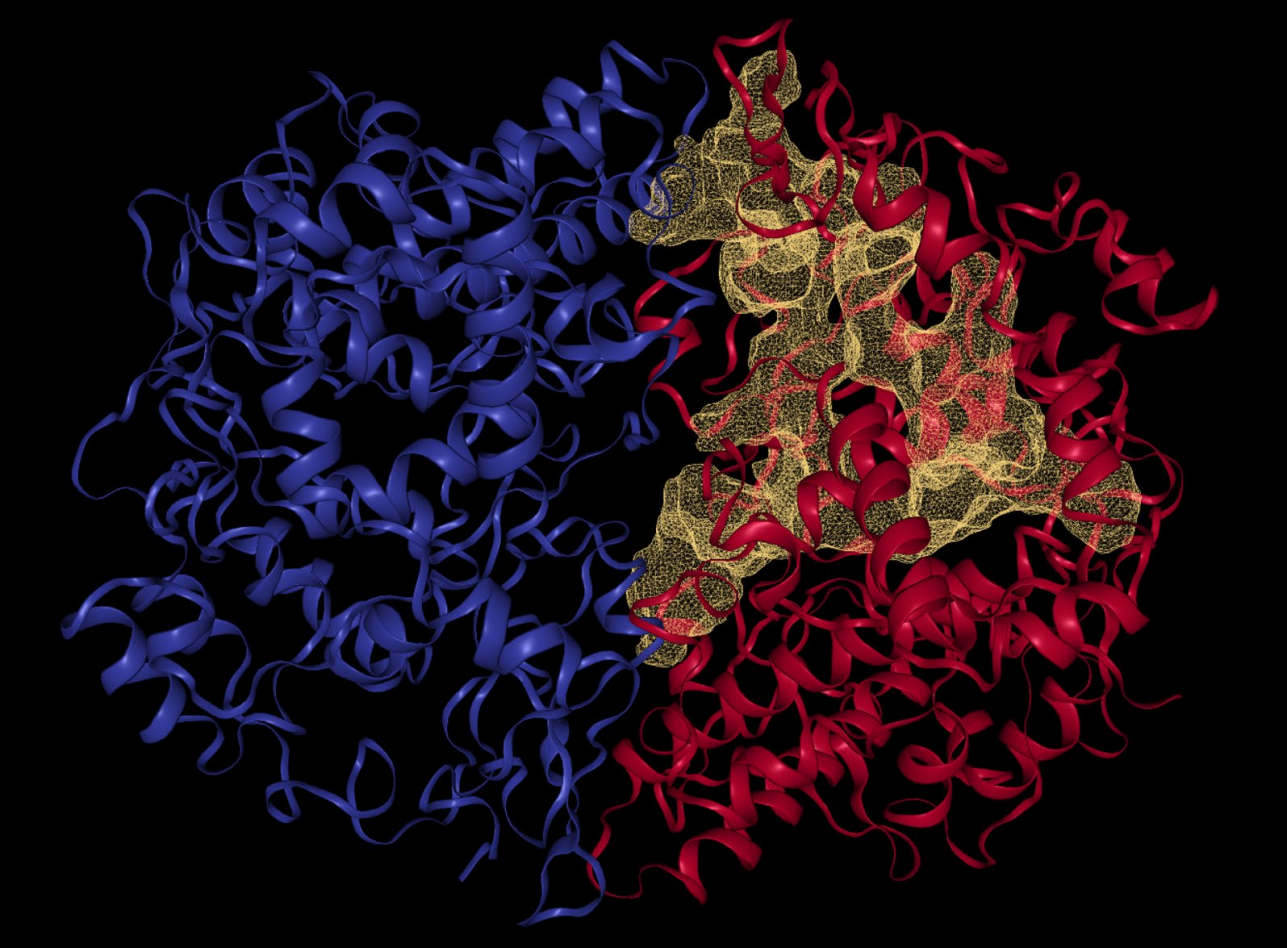
Optimal binding site of 5F1A determined by DoGSiteScorer

Protein active sites were predicted with the help of DoGSiteScorer [25]. The active site with the highest druggability (0.81) was chosen and cited in Fig. 2. AutoDock Vina 4.2 was employed to dock VCO components targeting important residues of COX-2 (VAL344, TYR348, VAL349, LEU384, TYR385, and TRP387). A grid-box for COX-2 receptor (PDB ID: 5f19) was created via AutoGrid4 using 50 Å ×50 Å ×50 Å with spacing of 0.5 Å as grid box dimensions, followed by centering the box around target residues, namely, VAL344, TYR348, VAL349, LEU384, TYR385, and TRP387. The target residues were according to Lucido et al. [7].

### Receptor–ligand docking

Lamarckian Genetic Algorithm together with the grid-based energy evaluation method was chosen for the molecular docking. Then, 50 Genetic algorithm runs were programmed on the docking software, followed by the population size set as 300 to get more significant results from the docking. The default settings were maintained for the other parameters and proceeded with docking. The best docking results were generated in dlg file type which was further analyzed and visualized by AutoDock Vina 4.2.

### Analysis and visualization

The best binding energy values of the ligands were visualized on AutoDock Vina 4.2 and saved as pdbqt file type. The files were then converted to pdb type via Open Babel software. The best docking pdb files were uploaded to protein–ligand interaction profiler (PLIP) (https://plip-tool.biotec.tu-dresden.de/plip-web/plip/index) and LigPlot to clearly visualize the binding position of the ligands with the proteins and its residues in 3D and 2D view, respectively.

### Molecular dynamic simulation

The molecular dynamic simulation (MDS) technique was used according to previous researchers with slight modifications [26, 27]. The best binding ligand–COX-2 complex was obtained upon molecular docking. The complex parameterization was done with CHARMM–GUI web interface (http://www.charmm-gui.org). To simulate physiological circumstances, the system was dissolved in a 10 Å waterbox using solution builder and neutralised with 0.15 molar NaCl. The isothermal–isobaric (NPT) was set with periodic boundary conditions. The temperature was 303.15 K and the pressure was held at 1 atm. The NAMD package was used to run the MD simulation using the CHARMM36 force field. The VMD programme was used to prepare the complex's simulation systems. After subjecting the system to a 10 ns equilibration period, the system followed through a 1000 frame of production run at 303.15 K. Hydrogen bonds were calculated via plotting all hydrogen bonds exhibited in the system against the 1000 conformations. The results for stability were analysed by plotting the RMSD values against the 1000 conformations utilizing RMSD trajectory of VMD. The results were saved in dat file type and plotted via QTGrace software. The results were proceeded to post-MDS redocking following previous protocols and visualized using Ligplot.

### AdmetSAR, Osiris and molinspiration calculations

Osiris and Molinspiration calculations are well-known and notable methods for creating 2D models to define and identify the how pharmacophore site affects biological properties when substitution of chemical occurs [28]. Any compound's pharmacological quality is determined by its drug-likeness or bioavailability, which is evaluated by its physicochemical properties. Thus, MCFAs (caproic acid, capric acid, caprylic acid, lauric acid, and myristic acid), MCMs (monolaurin, monocaprin, monocaproin, monocaprylin, and monomyristin), and reference drugs (aspirin, paracetamol, and ibuprofen) were computed for their physicochemical properties, solubility, and drug-likeliness via Osiris and Molinspiration calculator. The ADMET (Absorption, Distribution, Metabolism, and Excretion–Toxicity in pharmacokinetics issues) properties of VCO derivatives and the reference molecule were also predicted using the AdmetSAR online database [29].

## Results

### Pharmacophore visualization

The utilization of pharmacophore is to determine molecular similarity for alignment of ligand and protein structure and also to characterize the molecular frameworks that are essential for a specific biological property [30]. Pharmacophores are based on the chemical properties of functional groups which are definable as an aromatic (ARO), hydrogen donor (HD), hydrogen acceptor (HA), hydrophobic (HP), negative ion (NI), or positive ion (PI) via the pharmit server. Prior to molecular docking, pharmacophore data was utilised to identify compounds that match the query's structural and chemical functional requirements for COX-2 protein’s active pocket (Table 1).

**Table 1:** Pharmacophore of salicylic acid in crystal structure of 5F1A, reference drug, MCM and MCFA

Compound	Visualization	Pharmacophore
Salicylic acid (5F1A)		1HD 3HA 1HP 1NI 1ARO
Aspirin		4HA 2HP 1NI 1ARO
Ibuprofen		2HA 3HP 1NI 1ARO
Paracetamol		2HD 2HA 2HP 1ARO
Monocaproin		2HD 3HA 2HP
Monocaprylin		2HD 3HA 3HP
Monocaprin		2HD 3HA 4HP
Monolaurin		2HD 3HA 5HP
Monomyristin		2HD 3HA 5HP
Caproic acid		2HA 2HP 1NI
Caprylic acid		2HA 3HP 1NI
Capric acid		2HA 4HP 1NI
Lauric acid		2HA 5HP 1NI
Myristic acid		2HA 5HP 1NI
Aspirin		4HA 2HP 1NI 1ARO
Ibuprofen		2HA 3HP 1NI 1ARO
Paracetamol		2HD 2HA 2HP 1ARO

*ARO* denotes aromatic, *HD* denotes hydrogen donor, *HA* denotes hydrogen acceptor, *HP* denotes hydrophobic, *NI* denotes negative ion, and *PI* denotes positive ion

**Fig. 3:**
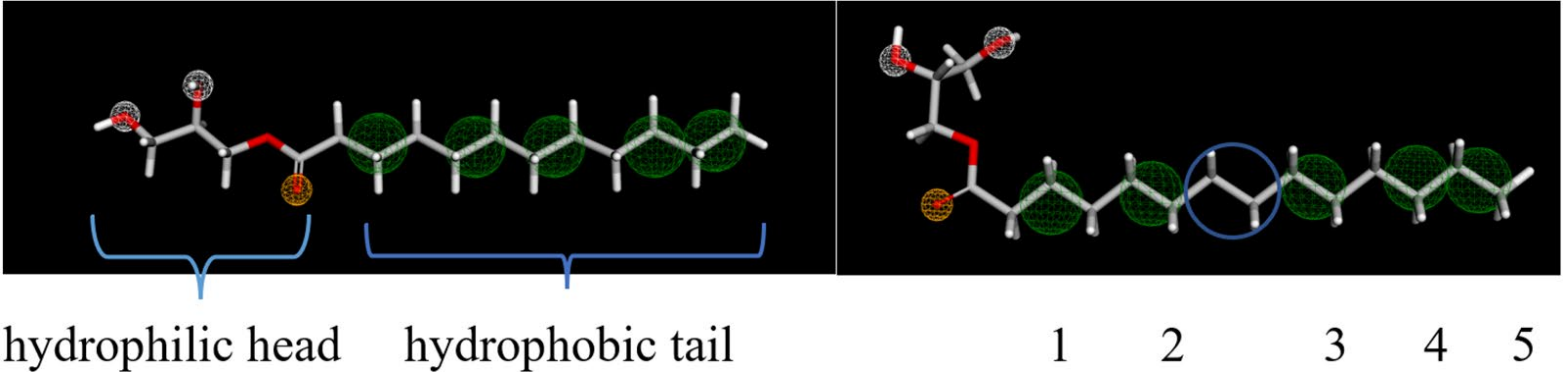
Structure and pharmacophore of monolaurin and monomyristin

MCMs exhibited three types of pharmacophore features, namely, HD, HA and HP. Monocaproin has two HD, three HA and two HP. Monocaprylin has two HD, three HA and three HP. Monocaprin has two HD, three HA and four HP. Monolaurin and monomyristin have two HD, three HA and five HP. The MCMs have same pharmacophores at the hydrophilic head with increasing HP proportional to the hydrophobic tail length. Monolaurin and monomyristin have similar pharmacophore features with monomyristin having a longer tail or space between the second and third HP pharmacophore, as shown in Fig. 3. MCFAs exhibited three types of pharmacophore features, namely, HD, HA and NI. Caproic acid has two HA, two HP and a NI. Caprylic acid has two HA, three HP and a NI. Capric acid has two HA, four HP and a NI. Lauric acid and myristic acid have two HA, five HP and a NI. The lauric acid have similar pharmacophore features as with myristic acid having a longer tail or space between the second and third HP pharmacophore. 2-Hydroxybenzoic acid or salicylic acid in 5f1a complex exhibited one HD, three HA, one HP, one NI and one ARO pharmacophore interactions. Aspirin has four HA, two HP, one NI and one ARO. Ibuprofen has two HA, three HP, one NI and one ARO. Paracetamol has two HD, two HA, two HP, and one ARO.

All the MCMs has three HA which compromise the HA of ibuprofen, paracetamol and salicylic acid but one HA less than aspirin. All the MCMs have two HD same as paracetamol; The presence of two HD is advantageous than aspirin and ibuprofen with zero HD followed by salicylic acid with only one HD. MCMs are lacking with NI and ARO which are present in all reference drugs but present with more HP for stable anchoring to receptor. Aspirin and paracetamol have two HP, while salicylic acid and ibuprofen have one and three, respectively. Monocaprin, monolaurin, lauric acid and capric acid with four to five HP are more beneficial for structure-based and docking-based studies due to the more HP for anchorage. All MCFAs have one NI same as the reference drugs but absent with any HD and having only two HA. Overall, monocaprin, monolaurin, lauric acid and capric acid showed the most suitable pharmacophore features compared to anti-inflammatory reference drug for further molecular docking studies.

### Molecular docking

Inflammatory disorders are currently a hot topic of study due to the harm they do to people's health all around the world. COX-2 is a human enzyme that regulates inflammation and is only expressed by the release of proinflammatory mediators. A set of bifunctional homodimeric enzymes, namely, COX-1 and COX-2 enzymes, catalysed arachidonic acid to produce prostaglandin (PG) H2 in the committed stage of PG biosynthesis [6]. Natural chemical derivatives from VCO can potentially inhibit and halt the pro-inflammatory signal expression by COX-2 with less side effects than classical NSAIDs (indomethacin aspirin, and ibuprofen) and inhibitors (celecoxib, rofecoxib, and valdecoxib) [5, 22].

In CADD, molecular docking has always been a popular tactic. The interaction between the target macromolecule such as enzyme, nucleic acid or receptor with the small molecule, namely, inhibitor, potential drugs or substrate, are being evaluated in docking studies [24]. The binding nature and the development of more effective medication candidates can be improved via this art. In this in silico study, VCO derivatives were docked against COX-2 using Auto Dock Vina 4.2. The COX-2 protein used for this investigation was retrieved in its 3D crystal structure (5f19), with the binding site surrounded by amino acids, such as VAL344, TYR348, VAL349, LEU384, TYR385, and TRP387. The fundamental step of molecular docking in drug design is to identify suitable fitting in the active pocket. For molecular docking, the lowest binding energy (kcal/mol) provides a fast estimation of a better or greater ligand–receptor complex binding affinity. Anticipation of the preferred orientation of the ligand when it is bound to the protein active pocket is possible utilizing the docking studies.

According to the docking results (Table 2), the binding score of MCMs fell in the range of − 7.02 to − 7.58 kcal/mol, while MCFAs obtained binding score falling in the range of − 5.62 to − 7.01 kcal/mol. The binding energy value displayed by the ligand, monolaurin obtained the utmost negative binding score in comparison with other MCMs and MCFAs when bound to COX-2 as the receptor with an energy binding affinity of − 7.58 kcal/mol. On the other hand, aspirin, ibuprofen, and paracetamol have also shown great binding results with the COX-2 receptor with energy binding affinity of − 7.14 kcal/mol, − 7.72 kcal/mol, and − 6.76 kcal/mol, respectively. In this case, a stronger anti-inflammatory effect is expected by the preferable binding of monolaurin (− 7.58 kcal/mol) to the COX-2 protein over other MCMs and MCFAs but less than ibuprofen (− 7.72 kcal/mol). The effect was also inferred from the higher number of 15 intermolecular interactions between the Monolaurin–COX-2 complex. As for aspirin, ibuprofen and paracetamol only having 6, 7 and 8 interactions which were outclassed by monolaurin with 15 interactions.

**Table 2:** Binding Scores and Interactions of Ligands Docked to COX-2

Ligands	Binding score (kcal/mol)	Hydrogen bond	Hydrophobic interactions	Total interactions
Monocaproin	− 7.47	PHE529, LEU531, GLY533, LEU534	LEU352, **TRP387, TRP387**, PHE518, PHE518, VAL523, VAL523	11
Monocaprylin	− 7.02	**TYR385**, **TYR385**, PHE529, LEU531, GLY533, LEU534	PHE381, **LEU384, TYR385, TRP387**, PHE518, PHE518, VAL523. VAL523	14
Monocaprin	− 7.56	GLN192, ARG513, ILE517, PHE518	LEU352, **LEU384, TYR385, TRP387,** PHE518, PHE518, VAL523	11
Monolaurin	− 7.58	**TYR385**, PHE529, LEU531, GLY533, LEU534	**VAL349,** LEU352, **LEU384, TYR385, TRP387,** PHE518, PHE518, VAL523, VAL523, ALA527	**15**
Monomyristin	− 7.53	VAL523	PHE205, PHE209, VAL228, **VAL349,** ILE377, PHE381, PHE381, LEU534, LEU534	10
Caproic	− 5.62	PHE529, LEU531, GLY533, LEU534	PHE205, PHE209, VAL228, ILE377, LEU534, LEU534	10
Caprylic	− 5.65	ALA527, LEU531	**LEU384, TYR385, TRP387,** VAL523	6
Capric	− 6.09	GLN192, ILE517, PHE518, HIS90 (salt bridge)	LEU352, **TRP387,** PHE518, VAL523, VAL523	9
Lauric	− 6.62	HIS90, GLN192, ILE517, PHE518	LEU352, PHE381, **LEU384, TYR385, TRP387,** PHE518, PHE518, VAL523, VAL523,	13
Myristic	− 7.01	GLY526, LEU531	LEU352, PHE381, **LEU384, TYR385, TRP387,** PHE518, PHE518, VAL523, VAL523, ALA527	12
Reference Drug				
Aspirin	− 7.14	**TYR385**, GLY526, LEU531	**PHE381, TRP387,** ALA527	6
Ibuprofen	− 7.72	LEU531, GLY533, LEU534	PHE205, **PHE381, TYR385**, LEU534	7
Paracetamol	− 6.76	**TYR385**, MET522, GLY526	**PHE381, PHE381, LEU384, TYR385, TRP387**	8

Bold texts indicate important residue at binding site of COX-2 protein, namely, VAL344, TYR348, VAL349, LEU384, TYR385, and TRP387

**Fig. 4:**
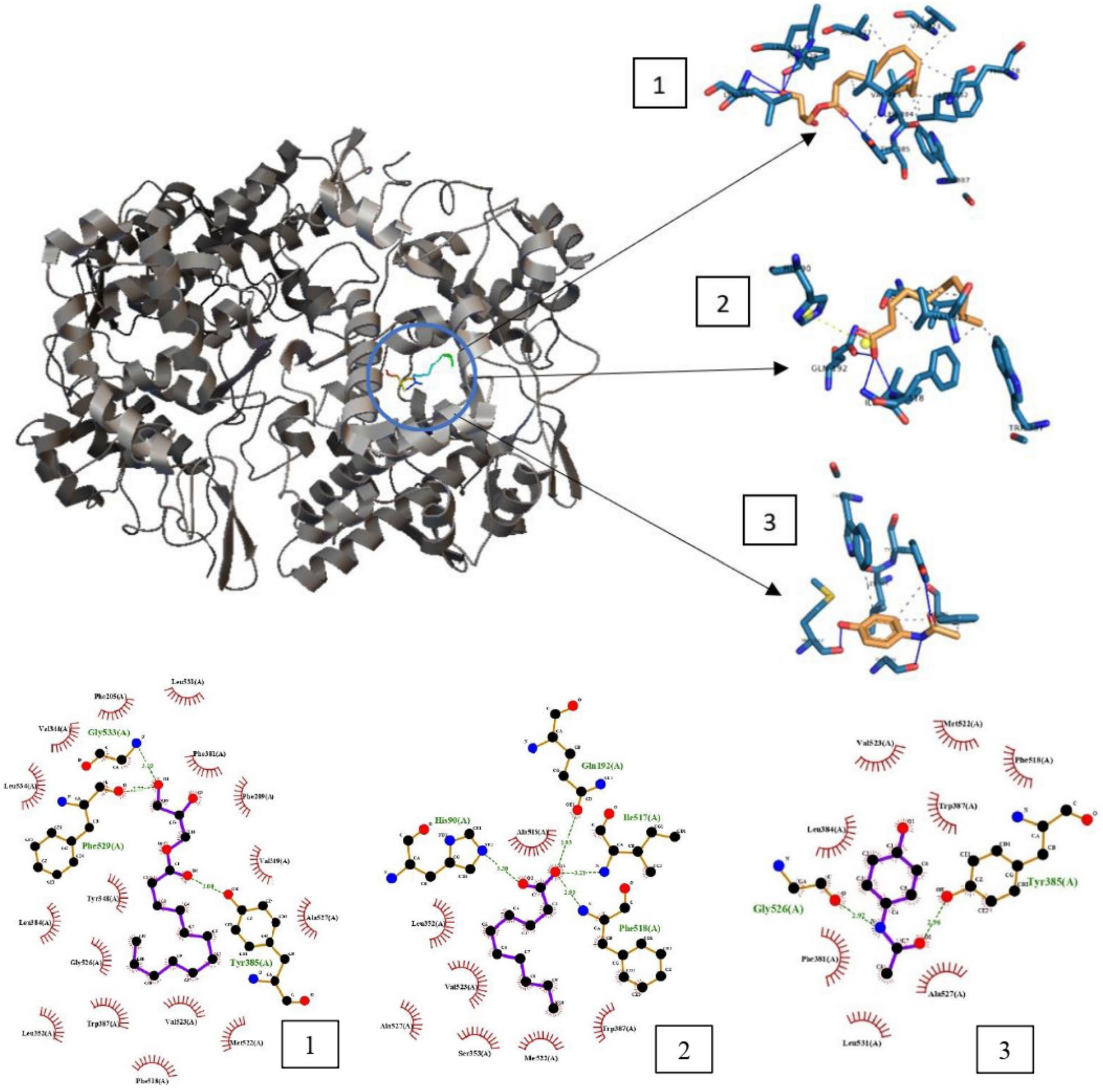
Crystal Structure of COX-2 protein with binding site occupied. (1) Monolaurin (2) Capric Acid and (3) Paracetamol. Blue/green lines indicates HB, dotted lines indicates HP, yellow dot and line indicates salt bridge formation

Furthermore, monolaurin with utmost negative binding score of− 7.58 kcal/mol and 15 interactions was prompt for binding pose visualization and bring to fore their interaction patterns with the target protein in relative to the reference drugs using PLIP. The ligand–receptor complex exhibited interactions, such as salt bridge formation, HP, and HB. First and foremost, a significant drug binding affinity is primarily due to strong HB between the drug and the receptor. The HB interactions between the protein and docked compounds were assessed to determine the stability of the best dock position of these compounds by highlighting the key residues engaged in hydrogen bond formation [7]. In general, HB are classified as strong if their distance is between 2.5 and 3.1 A, and weak if it is between 3.1 and 3.55 A [31, 32]. Stronger ligand–receptor complex is formed with more numbers of HB interacting with the targeted amino acid residue [33]. Based on the 3D model result via PLIP (Fig. 4), monolaurin was found forming HB with COX-2 protein such as the amino acid residues of TYR385, PHE529, LEU531, GLY533, and LEU534 with bond distance (BD) of 3.00, 2.77, 3.22, 3.10, and 3.38 in Å, respectively. Interaction with TYR385, PHE529, and GLY533 are classified as strong HB. All the HB with BD extracted from the dockings are cited in Table 3. HBs were mainly formed at the polar head of monolaurin. On the other hand, monocaprylin was detected to form the most HB with amino acid residues of TYR385, TYR385, PHE529, LEU531, GLY533, and LEU534 with BD (Å) of 4.10, 4.10, 2.71, 3.14, 3.05, and 3.88, respectively. However, the two HB formed with TYR385, main residue, are classified as weak HB, while only HB with PHE529, LEU531, and GLY533 are strong HB. Aspirin was found forming HB with COX-2 protein, such as the amino acid residues of TYR385, GLY526, LEU531 with BD (Å) of 2.88, 2.83, and 3.27, respectively, whereby TYR385 and GLY526 are strong HB. Ibuprofen was found forming HB with COX-2 protein, such as the amino acid residues of LEU531 (2.88 Å), GLY533 (2.95 Å), LEU534 (3.37 Å) with the absence of interaction with TYR385, the main residue. Paracetamol was found forming HB with COX-2 protein, such as the amino acid residues of TYR385, MET522, GLY526 with BD (Å) of 2.96, 2.85, and 2.92, respectively, whereby all the HB formed are strong.

**Table 3:** Hydrogen bond of ligands docked to COX-2

Ligands	Hydrogen Bond	Bond Distance (Å)
Monocaproin	PHE529 LEU531 GLY533 LEU534	**3.05** **2.96** **3.06** **3.08**
Monocaprylin	**TYR385** **TYR385** PHE529 LEU531 GLY533 LEU534	4.10 4.10 **2.71** **3.14** **3.05** 3.38
Monocaprin	GLN192 ARG513 ILE517 PHE518	**2.91** 3.18 **2.95** **2.89**
Monolaurin	**TYR385** PHE529 LEU531 GLY533 LEU534	**3.00** **2.77** 3.22 **3.10** 3.38
Monomyristin	VAL523	3.32
Caproic	PHE529 LEU531 GLY533 LEU534	**2.99** **2.93** **3.07** 3.15
Caprylic	ALA527 LEU531	**2.89** **2.93**
Capric	GLN192 ILE517 PHE518	**2.93** 3.21 **2.93**
Lauric	HIS90 GLN192 ILE517 PHE518	3.68 **2.89** 3.29 **2.99**
Myristic	GLY526 LEU531	**2.76** **2.76**
Reference drug		
Aspirin	**TYR385** GLY526 LEU531	**2.88** **2.83** 3.27
Ibuprofen	LEU531 GLY533 LEU534	**2.88** **2.95** 3.37
Paracetamol	**TYR385** MET522 GLY526	**2.96** **2.85** **2.92**

Bold texts indicate strong hydrogen bond (2.5–3.1A)

Hydrophobic interaction is a reaction that causes protein structures to compact to prevent any contact with water or aqueous conditions [34]. Nonpolar amino acid residues that cluster together forming groups in the protein interior to avoid the residues from interacting with water is another definition of hydrophobic interaction. Based on the 2D and 3D viewing via LigPlot and PLIP (Fig. 4), monolaurin displayed hydrophobic interactions with COX-2 protein, such as the amino acid residues of VAL349, LEU352, LEU384, TYR385, TRP387, PHE518, PHE518, VAL523, VAL523, ALA527. Hydrophobic interactions were mainly due to the hydrophobic tail of monolaurin. Aspirin was found forming hydrophobic interaction with COX-2 protein, such as the amino acid residues of PHE381, TRP387, and ALA527. Ibuprofen was found forming hydrophobic interaction with COX-2 protein, such as the amino acid residues of PHE205, PHE381, TYR385, LEU534. Paracetamol was found forming hydrophobic interaction with COX-2 protein, such as the amino acid residues of PHE381, PHE381, LEU384, TYR385, TRP387. In general, less hydrophobic interactions were exhibited by reference drug compared to monolaurin due to less flexibility of reference drug owing an aromatic group.

Besides hydrophobic interactions and hydrogen bonds, salt bridge might also play vital roles in the complex. Salt bridge formation is defined as having two ionized molecules, namely, one with a HB and the other with an electrostatic to react in a non-covalent way [35]. According to the 2D and 3D model generated, only capric acid formed salt bridge with COX-2 protein involving the amino acid residues of HIS90. However, none of the reference drugs have salt bridge interaction with any amino acid residues of the COX-2 protein despite having pharmacophore feature for NI. A clearer visualization of the best docking positions of capric acid and results are displayed in Fig. 4 and Table 2, respectively. In short, monolaurin elegantly exhibited the greatest docking score as a potent anti-inflammatory agent in comparison with the reference drugs.

To further validate the pharmacophore model descriptors, the poses and binding energies, and comprehensively investigate the interactions of the monolaurin within the catalytic site of the COX-2, MDS was conducted on the Monolaurin–COX-2 complex.

### Molecular dynamic simulation (MDS)

Atomic and molecular motions, as well as the system's evolution in a period of time, were investigated using MDS [20]. In MDS, hydrogen bonds (HB) are crucial for the formation of ligand–protein complex in an environment containing water as it may hydrolyse the system rendering it to break [36]. To establish a hydrogen bond, an electron negative member of an electronegative hydrogen-bond acceptor must bond with an electron positively charged member of an electropositive hydrogen-bond donor. The hydrogen bonds may perform as an attachment mechanism, specifying the spatial placement of the druggable molecules in the active pocket, promoting the HP and electrostatic interactions [37]. Furthermore, HB operates as a critical interaction in influencing the selectivity, affinity for binding and stabilising effect of the small molecules with the receptor.

**Fig. 5:**
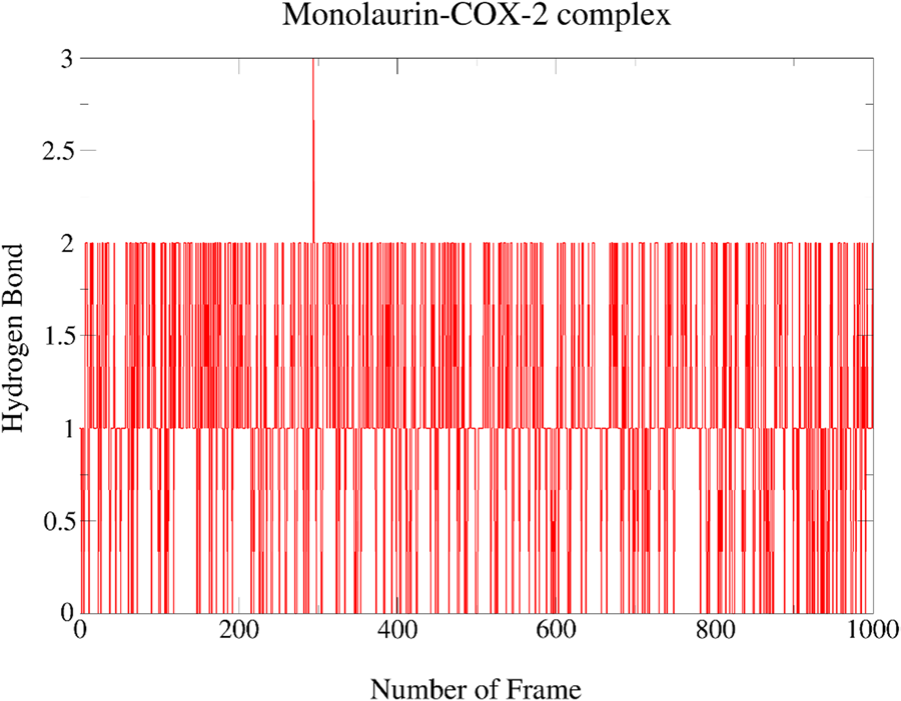
Hydrogen bonds plot representing 1000 frame of the simulation for Monolaurin–COX-2 complex

From Table 4 and Fig. 5, it was found that the trajectory of the Monolaurin–COX-2 complex shows the appearance of four HB between Monolaurin–COX-2 complex, out of which two H-bonds were observed to be long-living during the entire 1000 frames of simulation. Monolaurin was employed as H-bond acceptor to the side of residue TYR385 of COX-2 protein with the highest occupancy of 67.03%. It was also employed as H-bond donor to the side and main of residue PHE529 with occupancy of 46.75% and 0.30%, respectively. The residue LEU531 was also occupied by monolaurin with the least occupancy of 0.20%.

**Table 4:** Hydrogen bond of best docking complexes

Complex	HD	HA	Occupancy %
Monolaurin	Ligand-Side	PHE529-Side	**46.75**
	TYR385-Side	Ligand-Side	**67.03**
	Ligand-Side	PHE529-Main	0.30
	LEU531-Main	Ligand-Side	0.20
Monocaprylin	LEU531-Main	Ligand-Side	**50.75**
	Ligand-Side	TYR385-Side	2.10
	Ligand-Side	PHE529-Main	0.10
	LEU534-Main	Ligand-Side	0.30
	Ligand-Side	PHE529-Side	**34.07**
Aspirin	LEU531-Main	Ligand-Side	**21.28**
	PHE529-Side	Ligand-Side	0.20
	Ligand-Side	PHE529-Side	0.50
Paracetamol	TYR385-Side	Ligand-Side	**54.85**
	Ligand-Side	GLY526-Main	1.10
	Ligand-Side	MET522-Main	6.99
	Ligand-Side	PHE529-Side	0.60

Bold texts indicate the hydrogen bonds with highest occupancy formed by ligand with an active site residue

**Fig. 6:**
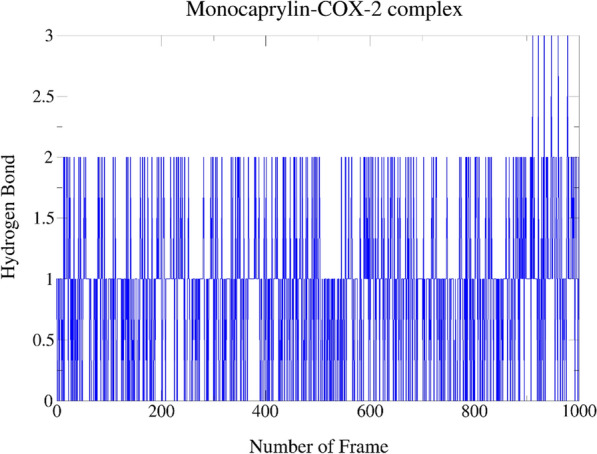
Hydrogen bonds plot representing 1000 frame of the simulation for Monocaprylin–COX-2 complex

According to data from Table 4 and Fig. 6, it was found that the trajectory of the Monocaprylin–COX-2 complex shows the appearance of four HB between Monocaprylin–COX-2 complex, with only one long-living H-bonds during the entire 1000 frames of simulation. Monocaprylin was employed as H-bond acceptor to the side of residue LEU531 of COX-2 protein with an occupancy of 50.73%. It was also employed as H-bond donor to the side and main of residue PHE529 with occupancy of 34.07% and 0.10%, respectively. It was further used as H-bond donor to the side of residue TYR385 with occupancy of only 2.10%. HB was also found between monocaprylin with main residue of LEU534 with monocaprylin as H-bond donor and an occupancy of 0.30%

**Fig. 7:**
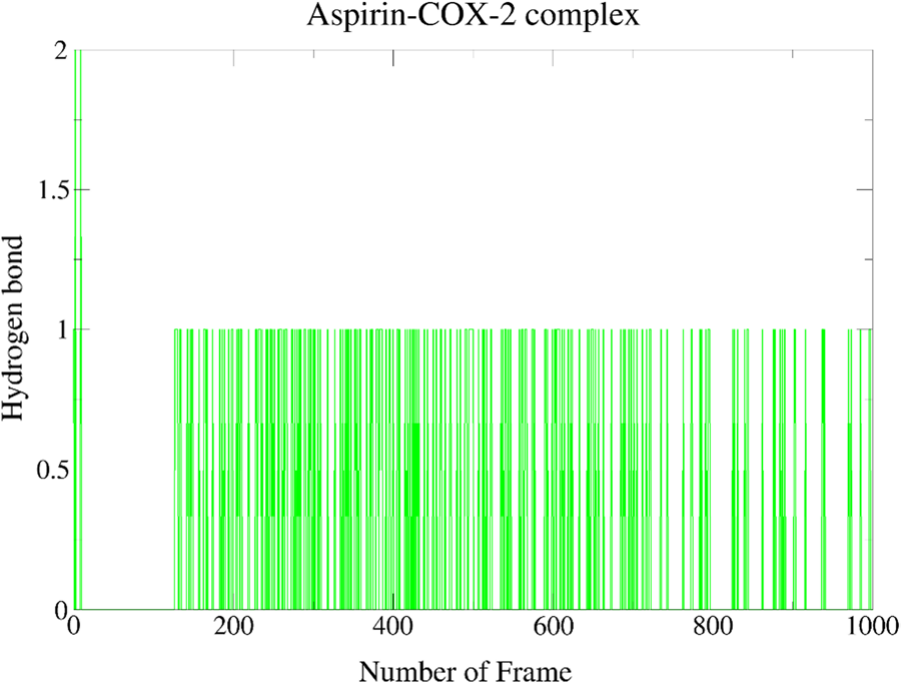
Hydrogen bonds plot representing 1000 frame of the simulation for Aspirin–COX-2 complex

Furthermore, we found that the trajectory of the Aspirin–COX-2 complex (Table 4 and Fig. 7) shows the appearance of three HB between Aspirin–COX-2 complex, with only one to be slightly long-living H-bonds during the entire 1000 frames of simulation. Aspirin was employed as H-bond acceptor to the main residue of LEU531 and the side residue of PHE529 with occupancy of 21.28% and 0.30%, respectively. It was employed as H-bond donor to the side of residue PHE529 of COX-2 protein with the occupancy of 0.50%. The HB of Aspirin with residue TYR385 were lost throughout the simulation.

**Fig. 8:**
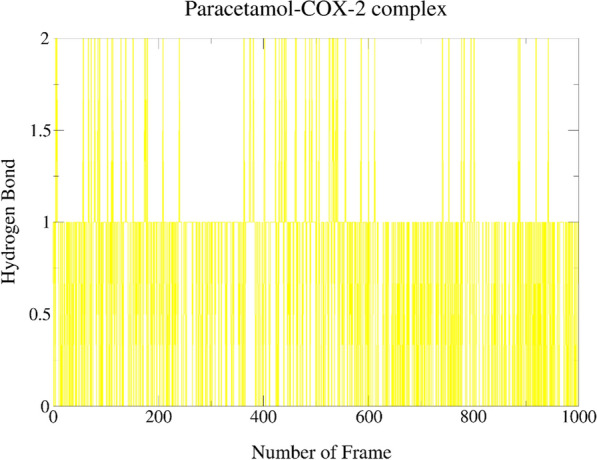
Hydrogen bonds plot representing 1000 frame of the simulation for Paracetamol–COX-2 complex

It was found that the trajectory of the Paracetamol–COX-2 complex (Table 4 and Fig. 8) shows the appearance of four HB between Paracetamol–COX-2 complex, with only one to be long-living H-bonds during the entire 1000 frames of simulation. Paracetamol was employed as H-bond acceptor to the side of residue TYR385 of COX-2 protein with a high occupancy of 54.85%. It was also employed as H-bond donor to the side residue of PHE529, main residue of GLY526 and main residue of MET522 with occupancy of 0.60%, 1.10% and 6.99%, respectively.

**Fig. 9:**
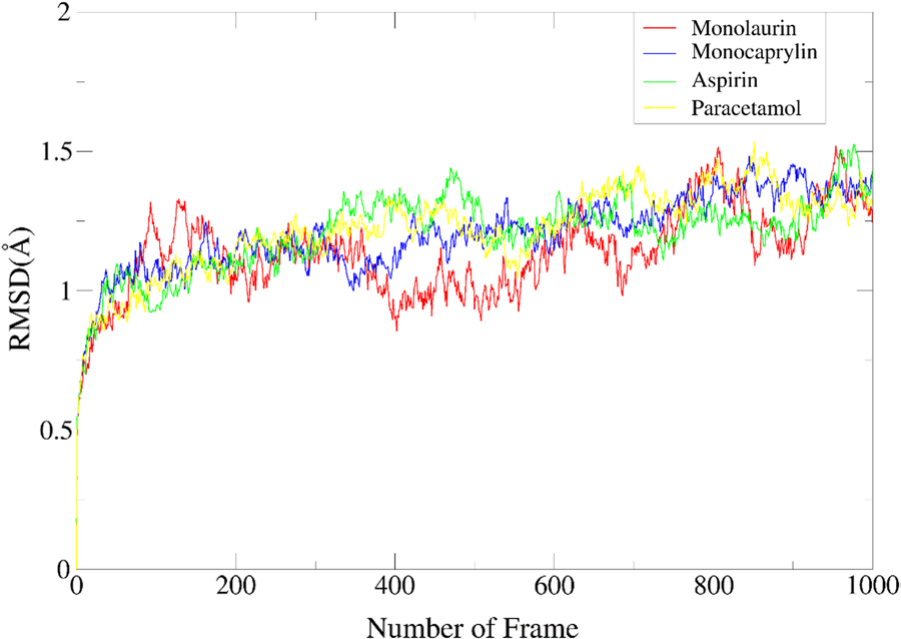
Average RMSD plot representing 1000 frame of the simulation for the best docking complexes. Red for Monolaurin–COX-2 complex, blue for Monocaprylin–COX-2 complex, green for Aspirin–COX-2 complex and yellow for Paracetamol–COX-2 complex

To better understand the stability and dynamical behaviour of the Monolaurin–COX-2 complex over a period, classical MD simulations were conducted. The root mean square deviation (RMSD) is a measure of the protein–ligand complex's conformational changes and stability [37]. Thus, obtaining a low value for RMSD indicate the Monolaurin–COX-2 complex is stable and vice versa [36]. It is also crucial to take note that a low value of RMSD (< 2 Å) indicates significant binding affinity and the establishment of a stable complex [26]. Backbone RMSD calculations were performed to describe the changes in conformational of the best docking complexes with result shown in Table 5 and Fig. 9.

**Table 5:** RMSD scores of best docking complexes

Complex	Average	Standard deviation	Minimum	Maximum	Redock score
Monolaurin	1.137	0.153	0.480	1.520	− 4.87
Monocaprylin	1.206	0.134	0.501	1.483	− 4.45
Aspirin	1.204	0.138	0.548	1.526	− 7.37
Paracetamol	1.220	0.149	0.501	1.535	− 5.57

The lowest RMSD value of 1.137 $\pm$ 0.153 Å was exhibited by the Monolaurin–COX-2 complex and falling in the range of 0.480–1.520 Å indicating the strong binding of the complex formed. Monocaprylin–COX-2 complex showed great RMSD score of 1.206 $\pm$ 0.134 Å and ranged in 0.501–1.483 Å, while Aspirin–COX-2 complex showed RMSD score 1.204 $\pm$ 0.138 Å and ranged in 0.548–1.526 Å. Higher RMSD value of 1.220 $\pm$ 0.149 Å was exhibited by the Paracetamol–COX-2 complex and falling in the range of 0.501 to 1.535 Å. Overall, all small molecules formed stable complexes with COX as indicated by the average RMSD of all complexes falling below 2 Å. The stability of the ligand within the active site of the receptor can also be represented through fluctuations in the RMSD plot of the ligand [21]. In this case, all ligand-complexes showed a consistent and steady performance throughout the entire 10 ns simulation, indicating the ligand complex stability within the system. The plot for Monolaurin–COX-2 complex exhibited slight fluctuations compared to other complexes but not determined as large or extreme fluctuations which imply major movement of the ligand from the receptor pocket during simulation. Overall, all small molecules formed stable complexes with COX-2 as indicated by the average RMSD of all complexes falling below 2 Å and acceptable fluctuation of the RMSD plot.

### Post-MDS Analysis

According to the post-MDS docking results (Tables 5, 6), binding score of monolaurin, monocaprylin and paracetamol fell from − 7.58, − 7.02 and − 6.76 to − 4.87, − 4.45 and − 5.57 in kcal/mol from the pre-MDS results, respectively. Aspirin shown increment in binding energy from − 7.14 to − 7.37 kcal/mol. Nevertheless, monolaurin maintained the most HB (MET522, VAL523, and TYR385) as compared to monocaprylin and reference drugs (zero HB) which is depicted in Table 6. Only monolaurin maintained the hydrogen bond with TYR385 (2.87 Å) which was relatively strong despite the drop in binding affinity. Although the binding score of aspirin was more favourable, no HB was formed upon the post-MDS redocking. Overall, the result showed that the Monolaurin–COX-2 complex's conformational integrity was considerably significant after MDS, with the ligand remaining securely bound with the main residue within the active cleft of the COX-2 protein upon a 10 ns simulation and redocked.

**Table 6:** Active site of pre and post MDS

Ligand	Pre MDS	Post MDS
Monolaurin		
Monocaprylin		
Aspirin		
Paracetamol		

### AdmetSAR, Osiris and molinspiration calculations

To predict the toxicity of screened substances, the US Food and Drug Administration's toxicity risk calculator software, namely, Osiris and the AdmetSAR server, can be utilized [39]. While the structure-based design is becoming more common, many potential drugs are still unable to enter clinics due to ADMET. For instance, the cytochromes P450 enzyme is a well-known class of enzymes that are known for many ADMET issues [40]. Many adverse drug reactions may occur when these enzymes are inhibited or when undesirable metabolites are produced.

Osiris and Molinspiration calculations are well-known and notable methods for creating 2D models to define and identify the how pharmacophore site affects biological properties when substitution of chemical occurs [28]. Any compound's pharmacological quality is determined by its drug-likeness or bioavailability, which is evaluated by its physicochemical properties. Thus, components of VCO and reference drugs were computed for their ADMET, physicochemical properties, solubility, and drug-likeliness via AdmetSAR, Osiris and Molinspiration calculator.

### Osiris calculation analysis

The Drug Score (DSC) was included with physicochemical properties, namely, toxicity risks, drug-likeness, cLogP, solubility, and molecular weight in an organized system of values utilized to evaluate a substance’s potential as a drug candidate. Moreover, the Osiris program was able to calculate different properties of toxicity risk, for example, reproductive development toxicity (RT), tumorigenicity (T), mutagenicity (M), and irritation (I). The values obtained from the Osiris program for VCO components followed by reference molecules are recorded in Table 7. The DSC ranges from 0 to 1, with 1 indicating that the molecule has a reasonable chance of becoming a good drug choice and 0 indicating that a compound has no chance of becoming a drug molecule [41]. Most of the marketed drugs are reported with Solubility (logS) number falling in the range between − 4 and − 2.

**Table 7:** Osiris calculation of MCM, MCFA and reference drug

Compound	Osiris calculations					
	cLogP	Solubility	MW	Drug-likeliness	Drug score	Toxicity
Monolaurin	3.59	− 2.95	274	− 25.4	0.41	–
Monocaprin	2.68	− 2.41	246	− 25.4	0.45	–
Monocaprylin	1.77	− 1.87	218	− 25.52	0.47	–
Monocaproin	0.86	− 1.33	190	− 15.74	0.17	–
Monomyristin	4.49	− 3.49	302	− 25.44	0.35	–
Caproic acid	1.52	− 1.54	116	− 13.09	0.17	M, I
Caprylic acid	2.43	− 2.08	144	− 25.32	0.17	M, I
Capric acid	3.34	− 2.62	172	− 25.22	0.16	M, I
Lauric acid	4.24	− 3.16	200	− 26.22	0.08	M, T, I
Myristic acid	5.15	− 3.70	228	− 25.22	0.12	M, I
Reference molecule						
Aspirin	1.13	− 1.93	180	− 0.48	0.14	M, T, R
Ibuprofen	3.00	− 2.89	206	3.97	0.31	M, R
Paracetamol	1.02	− 1.66	151	1.93	0.20	M, T, R

clogP: LogP (octanol/water partition coefficient), MW: Molecular Weight, M: Mutagenic, I: Irritant, T: Tumorigenic, R: Reproductive effective, no toxicity: -

Thus, only capric acid, caprylic acid, lauric acid, myristic acid, monolaurin, monocaprin, monomyristin, and ibuprofen are complied with a good range of logS. These compounds are notable to have remarkable solubilizing properties and potential for better anti-inflammatory activity. Based on their small size (low MW) and solubility, this would also indicate a preferable drug delivery ability for the compounds [42]. In terms of DSC, MCFA did not showed promising DSC with a range of 8–17%. Interestingly, the MCM, namely, monolaurin, monocaprin, and monocaprylin, showed low moderate DSC of 41%, 45%, and 47%, respectively. The reference molecules also exhibited lower DSC, such as aspirin (14%), ibuprofen (31%), and paracetamol (20%) as compared with the aforementioned MCM. Overall, the MCM particularly monolaurin, monocaprin, and monocaprylin shown excellent drug physicochemical properties than MCFA and even the reference drugs (aspirin, ibuprofen, and paracetamol) confirmed by Osiris calculations.

### Molinspiration calculation analysis

Drugs are categorized depending on their drug-likeness property using Lipinski's rule of five (RO5), otherwise an empirical rule of thumb in determining the drug-likeliness of molecules with smaller molecular weight (MW) [43]. According to RO5, oral drug molecules are preferred to have a cLogP of not more than 5, a MW of not more than 500, not more than 10 hydrogen bond acceptors, and not more than 5 hydrogen bond donors [41, 43]. The bioactivity score was calculated for GPCR ligand, ion channel modulator (ICM), kinase inhibitor (KI), nuclear receptor ligand (NRL), protease inhibitor (PI), and enzyme inhibitor (EI). The physicochemical properties (MW, clogP, TPSA, ON, OHNH, Violations, Robust, Volume, GPCR/L, ICM, KI, NRL, PI, and EI) of MCMs, MCFAs and reference molecules are valued in Table 8. The value of bioactivity score (GPCR/L, ICM, KI, NRL, PI, and EI) that is greater than 0.00 indicated the molecule is active, less than − 0.50 is indicated as inactive, and between − 0.50 and 0.0 is indicated as fairly active [41].

**Table 8:** Molinspiration calculation of MCM, MCFA and reference drug

Compound	Molinspiration Calculation								Drug score					
	MW	clogP	TPSA	ON	OHNH	RO5	Robust	Volume	GPLP/L	ICM	KI	NRL	PI	EI
Monolaurin	274.40	4.07	66.76	4	2	0	14	291.65	0.08	0.05	− 0.19	− 0.00	− 0.02	0.27
Monocaprin	246.35	3.06	66.76	4	2	0	12	258.05	− 0.03	0.04	− 0.34	− 0.14	− 0.15	0.24
Monocaprylin	218.29	2.05	66.76	4	2	0	10	224.44	− 0.17	− 0.01	− 0.53	− 0.32	− 0.31	0.18
Monocaproin	190.24	1.04	66.76	4	2	0	8	190.84	− 0.35	− 0.10	− 0.78	− 0.54	− 0.51	0.10
Monomyristin	302.45	5.08	66.76	4	2	1	16	325.25	0.14	0.04	−0.09	0.08	0.07	0.26
Caproic acid	116.16	2.01	37.30	2	1	0	4	123.40	− 2.65	− 2.46	− 3.49	− 2.56	− 2.67	− 2.31
Caprylic acid	243.35	3.17	66.40	4	2	0	11	255.60	0.06	0.01	− 0.55	− 0.21	0.06	0.17
Capric acid	172.27	4.03	37.30	2	1	0	8	190.61	− 0.46	− 0.14	− 1.03	− 0.45	− 0.56	− 0.07
Lauric acid	200.32	5.04	37.30	2	1	1	10	224.22	− 0.27	− 0.04	− 0.75	− 0.24	− 0.36	0.04
Myristic acid	228.38	6.05	37.30	2	1	1	12	257.82	− 0.11	0.03	− 0.51	− 0.06	− 0.19	0.13
Reference molecule														
Aspirin	180.16	1.43	63.60	4	1	0	3	155.57	− 0.76	− 0.32	− 1.06	− 0.44	− 0.82	− 0.28
Ibuprofen	206.28	3.46	37.30	2	1	0	4	211.19	− 0.17	− 0.01	− 0.72	0.05	− 0.21	0.12
Paracetamol	151.16	0.68	49.33	3	2	0	1	140.1	− 1.05	− 0.54	− 1.04	− 1.21	− 1.20	− 0.68

*ClogP* LogP (octanol/water partition coefficient), *RO5* rules of 5, *GPLP/L* GPCR ligand, *ICM* ion channel modulator, *KI* kinase inhibitor, *NRL* nuclear receptor ligands, *PI* protease inhibitor, *EI* enzyme inhibitor

Utilizing Molinspiration, cLogP, namely, octanol per water partition coefficient or lipophilicity, was computed as an overall correction factor and fragment contributions. This system is highly reliable, and it can handle almost any organometallic and organic molecule. The cLogP values for MCFAs ranged in 2.01–6.05, while MCMs ranged in 1.04–5.08. Lauric acid, myristic acid, and monomyristin demonstrated clogP values of more than 5, whereby the absorption or permeation is low in the human body. The high clogP values of lauric acid, myristic acid, and monomyristin are mainly due to the long hydrophobic ends. In accordance, cLogP is inversely proportional with the absorption or permeation, for instance, upon 5 or a higher cLogP, absorption or permeation is reduced [44].

The Molecular Polar Surface Area (TPSA) from Molinspiration is measured using a published methodology and it is based on an overall fragment-based contribution, with O- and N-dependent polar moieties [45]. TPSA has been demonstrated as a notable and remarkable measurement in the absorption of drugs, namely, Caco-2 permeability, bioavailability, blood–brain barrier penetration, and intestinal absorption [40, 46]. All the tested compounds showed good TPSA values without exceeding 160. According to Rachedi et al. compounds having TPSA of 160 or higher are likely to be poorly absorbed in the intestine [46]. Thus, it is notable that TPSA and cLogP number are two significant physicochemical parameters for determining the absorption of an oral drug, yet they are not adequate data to draw a final conclusion [28].

In short, the MCFA and MCM such as caproic acid, capric acid, caprylic acid, monolaurin, monocaprin, monocaproin, and monocaprylin shown excellent physicochemical properties compatible with reference drugs (aspirin, paracetamol, and ibuprofen) confirmed via Molinspiration calculations.

### AdmetSAR calculation analysis

The analysis include in the ADMET study are, namely, blood–brain barrier (BBB) penetration, Caco-2 permeability, human intestinal absorption (HIA), P-glycoprotein inhibitor/substrate, human ether-a-go-go-related gene (hERG) inhibitors, CYP450 inhibitor/substrate, carcinogens, AMES mutagenicity, honeybee toxicity, fish toxicity, tetrahymena pyriformis toxicity, aqueous solubility and rat acute toxicity as cited in Table 9.

**Table 9:** ADMET calculation of MCM, MCFA and reference drug

ADMET predicted profile—classification							
Model	Result	Probability					
		MCM	MCFA	Caproic	Aspirin	Paracetamol	Ibuprofen
Absorption							
Blood–brain barrier	BBB +	0.5794	0.9488	0.9657	0.9376	0.9544	0.9619
Human intestinal absorption	HIA +	0.9310	0.9888	0.9867	0.9645	0.9921	0.9927
Caco-2 permeability	Caco2-	0.6062	0.8326	0.8092	0.6607	0.8285	0.8866
P-glycoprotein substrate	Substrate	0.6014	0.6321	0.6103	0.6850 (non)	0.8202 (non)	0.7590 (non)
P-glycoprotein inhibitor	Non-inhibitor	0.8441	0.9598	0.9618	0.9118	0.9820	0.9705
	Non-inhibitor	0.7798	0.9277	0.9651	0.9615	0.9781	0.9323
Renal organic cation transporter	Non-inhibitor	0.9135	0.9266	0.9360	0.9140	0.9292	0.9323
Distribution							
Subcellular localization	Mitochondria	0.7898	0.5152	0.5379	0.9369	0.7695	0.6974
Metabolism							
CYP450 2C9 Substrate	Non-substrate	0.8889	0.7886	0.7728	0.7518	0.7259	0.7594
CYP450 2D6 substrate	Non-substrate	0.8304	0.8956	0.8966	0.9116	0.8918 (sub)	0.9116
CYP450 3A4 substrate	Non-substrate	0.6858	0.6982	0.6955	0.7225	0.5554	0.6877
CYP450 1A2 inhibitor	Non-inhibitor	0.6657	0.8326	0.8055 (inhibitor)	0.9046	0.9045	0.9045
CYP450 2C9 inhibitor	Non-inhibitor	0.8948	0.8808	0.8977	0.9071	0.9070	0.9305
CYP450 2D6 inhibitor	Non-inhibitor	0.9128	0.9554	0.9504	0.9576	0.9755	0.9231
CYP450 2C19 inhibitor	Non-inhibitor	0.8693	0.9578	0.9474	0.9445	0.9161	0.9881
CYP450 3A4 inhibitor	Non-inhibitor	0.8425	0.9484	0.9531	0.9611	0.8496	0.9655
CYP inhibitory promiscuity	Low CYP Inhibitory Promiscuity	0.9642	0.9647	0.9587	0.9557	0.8842	0.9691
Excretion							
Toxicity							
Human ether-a-go-go-related gene inhibition	Weak inhibitor	0.9652	0.9322	0.9515	0.9433	0.9717	0.9719
	Non-inhibitor	0.6466	0.8868	0.8971	0.9799	0.9597	0.9734
AMES toxicity	Non-AMES toxic	0.9132	0.9865	0.9850	0.9326	0.8767	0.9894
Carcinogens	Non-carcinogens	0.7897	0.6452	0.6359	0.8356	0.7654	0.5553 (carcinogen)
Fish toxicity	High FHMT	0.8950	0.9144	0.7928	0.9391	0.6816	0.9471
Tetrahymena pyriformis toxicity	High TPT	0.9891	0.9990	0.9766	0.8519	0.6528	0.9961
Honey bee toxicity	High HBT	0.6262	0.6691	0.6652	0.7453	0.6871	0.7451
Biodegradation	Ready biodegradable	0.9480	0.8795	0.8962	0.9067	0.6342	0.5142
Acute oral toxicity	IV	0.6566 IV	0.6378 IV	0.8226 III	0.7260 II	0.8429 III	0.8084 III
Carcinogenicity (Three-class)	Non-required	0.7095	0.7057	0.6773	0.7139	0.4806 (warning)	0.7313

ADMET predicted profile—regression							
Model	Value	Unit					
Absorption							
Aqueous solubility	LogS	− 2.0181	− 3.5022	− 2.0177	− 1.7826	− 1.1313	− 3.9041
Caco-2 permeability	LogPapp, cm/s	0.0083	1.3950	1.4589	0.5054	1.5175	1.7486
Distribution							
Metabolism							
Excretion							
Toxicity							
Rat acute Toxicity	LD50, mol/kg	0.8172	1.3275	1.6604	2.6386	1.8596	2.3092
Fish toxicity	pLC50, mg/L	2.5412	1.8920	2.6304	− 0.1352	2.7860	1.3122
Tetrahymena pyriformis toxicity	pIGC50, ug/L	0.8291	0.3852	− 0.3351	0.2236	− 0.6949	1.3858

All compounds have satisfactory BBB and HIA scores, as determined by the AdmetSAR server's ADMET attributes. If the HIA is high, then the substance may be more readily absorbed by the body after being taken orally [47]. Aside from ibuprofen, all VCO compounds and the reference drugs are non-carcinogenic. All of the derivatives of VCO showed only mild inhibition of Herg, whereby long QT syndrome could be the result of hERG inhibition [19]. Predictions of efflux by P-glycoprotein (PGP) showed that MCM and MCFA are substrates and noninhibitors of PGP, respectively, while all reference medicines are nonsubstrate and noninhibitor of PGP. When blocked, drugs are unable to be absorbed, transported across cell membranes, or affects the drug retention.

Metabolismwise, it was discovered that each of the compound tested are non-inhibitor of CYP450 except for caproic acid which was an inhibitor to CYP450 1A2. Then, paracetamol was the only substrate for CYP 2D6 among the compound computed. When a molecule is deemed to be a noninhibitor of CYP450, it does not interfere with the bioconversion of medications that are metabolised by the CYP450 enzyme [47]. The antioxidant capacity of VCO may be linked to its hepatoprotective action, which is hypothesized through VCO derivatives does not take part in CYP450 system modulation [48]. To ascertain whether or not a substance was mutagenic, the AMES toxicity test was computed. All the tested compounds, including the reference drugs, showed no toxicity via series of tests deeming them as non-mutagenic. Except for caproic acid, all MCM and MCFA were indicated as IV category acute oral toxicity; ibuprofen, paracetamol, and caproic acid were indicated as III category acute oral toxicity; aspirin was revealed as II category acute oral toxicity. In addition, the median lethal dosage (LD50) values for all VCO derivatives are lower than those of the reference medications when tested on for rat acute toxicity. In a nutshell, VCO derivatives are positively harmless than the reference drugs via the ADMET tests.

## Discussion

Conventional medicinal development procedures are time-consuming and expensive, leading to the development of cost-effective and quick computational techniques for the creation and screening of potent drugs for clinical use [30]. Drugs passing the green light may be repurposed as an alternate means of screening for the rapid detection of probable lead sources to an disorder [37]. Recently, new in-silico investigations on medicinal plants, drug design, and vaccine development have arisen [49–51]. With the combination of pharmacological therapy, natural products can be beneficial as treatment to various illness [52]. However, some potential natural drugs are not found in the market due to the limitation for large scale production for instance marine sponges. However, VCO is already established by extraction and purification and can be produced in bulk as readily available supplement and healthy food oil.

Inflammatory diseases are important research issues right now due to the impact they bring to people's health all around the world. A human enzyme called COX-2 controls inflammation and is only activated when proinflammatory mediators are secreted. In the committed stage of PG biosynthesis, COX-1 and COX-2, two homodimeric bifunctional enzymes, catalysed the formation of PG H2 from arachidonic acid [6]. Arachidonic acid also takes part in the formation of pro-inflammatory lipid mediators including leukotrienes, which are activated by 5-lipoxygenase-activating protein, as well as anti-inflammatory mediators such as epoxyeicosatrienoic acids and followed by the hydrolysis of soluble epoxide hydrolase [17]. Therefore, inhibiting the two enzymes particularly COX-2 appears to be a potential strategy for regulating arachidonic acid-derived inflammatory mediators. In contrast to aforementioned standard NSAIDs and inhibitors, natural chemical compounds from VCO may be able to block and stop COX-2 from expressing pro-inflammatory signals with less side effects.

The MCT derivatives of VCO such as MCMs and MCFAs possesses versatile pharmacological ability that can help in immune system, treating various disorders including inflammation and have the capability to fight off numerous microbial infections [3]. For further approvals, in vivo validation studies have shown that distinct VCO preparations, particularly fermented VCO, have great antioxidant potentials [53–55]. VCO's anti-inflammatory and immunomodulatory properties have also been studied in a variety of preclinical animal models [10, 11, 13, 56].

From the pharmacophore visualization, monolaurin and monomyristin among the MCMs have the better pharmacophore features, namely, having two HD, three HA and five HP. Monolaurin and monomyristin have similar pharmacophore interactions with monomyristin having a longer tail or space between the second and third HP interactions. These pharmacophore features are more than sufficient to satisfy the one HD, three HA, and one HP interaction shown by salicylic acid in the 5f1a complex. It is interesting to state that the hydrophilic head of MCMs have overlapping HD and HA for the first two pharmacophores (white balls in Fig. 3) which act depending on the receptor conditions. This may also be a limitation as the overall hydrogen bonds formed are three instead of five despite having two HD and three HA. All the MCMs exhibited more HD than reference drugs but having one HA less than aspirin. Monolaurin and monomyristin exhibited more HP than all the reference drugs. All the MCMs also has three HA which compromise the HA of ibuprofen, paracetamol, and salicylic acid but one HA less than aspirin. A pharmacophore study was done by Ruslin et al. (2022) based on (R)-naproxen to screen for COX-2 inhibitor via the ZINC database. (R)-naproxen has seven pharmacophores, namely, three HP and HA with an NI. This includes the naphthalene ring's HP and NI features, followed by the carboxyl group's HA feature of the (R)-naproxen moieties. These pharmacophores are noted to have significant effects on COX-2 inhibition. In this case, MCMs, namely, monolaurin and monomyristin, have shown compatible pharmacophore features with (R)-naproxen with two more HP and HD, while NI feature are available in all MCFA. Pharmacophore-based and docking-based molecular modelling approaches are more advantageous when apply simultaneously. Some of the disadvantages of the two approaches may be mitigated this way, allowing for more effective outcomes.

In general, scoring functions in Autodock are broken down into three parts, namely, empirically, knowledge-based and force field-based. These functions are used to rank, categorize, and score the binding affinities of the many generated poses in docking [57]. Developing reliable scoring functions for molecular docking has been difficult. As the growing importance of accurately estimating binding affinities in drug design is unforeseeably gaining interest, leading to quantum mechanical (QM) approaches to become incredibly popular for designing drugs [58]. It has been discovered that certain computation of QM parameters, such as a dipole moment and atomic charges, can help increase the precision of a docking process [58, 59]. According to the theory of Gibbs’s free energy, the higher or more negative the *Δ*G value energy produced from the bonds between the ligands and the receptors, the more stable are the bonds [60]. The higher or more negative energy released from the ligand and receptor interactions, the more powerful the ligands adhere to the receptor. This is because ligand–receptor complex forms stable and strong non-covalent interactions which creates an opening to the cell leading to cell destruction as it interrupts cell metabolism and the replication of DNA [34]. In a very simple way, the *Δ*G value energy or the negativity of binding affinity is directly proportional to the stability of ligand–receptor complex formation. This also explained the fundamentals of molecular docking, whereby why the lowest binding energy (kcal/mol) provides a better or greater ligand–receptor complex binding affinity.

Interactions revealed the formation of HP, HB and salt bridges between proteins and ligands in silico study, which affects the binding energy affinity value of the small molecules in the complex [61]. According to the docking results, the binding energy value displayed by the monolaurin obtained the best binding score (− 7.58 kcal/mol) in comparison with other MCMs and MCFAs when bound to COX-2 receptor. In this case, a stronger anti-inflammatory effect is expected by the preferable binding of monolaurin (− 7.58 kcal/mol) to the COX-2 protein over other MCMs and MCFAs expect ibuprofen (− 7.72 kcal/mol). Therefore, more conclusions can be drawn from the interaction profiles of the ligands, namely, monolaurin having 15 intermolecular interactions between the COX-2 complex while ibuprofen only having 7 interactions. Despite ibuprofen having slightly higher binding score than monolaurin, the interaction formed will be less stable as compared to monolaurin. It is interesting to mention that monolaurin and monomyristin have different dock scores despite having the same pharmacophore features. This is due to their difference in spatial arrangement of the two small molecules with monolaurin having a better pharmacophore feature arrangement without any empty space.

From 2 and 3D diagram generated by Ligplot and PLIP (Fig. 4), monolaurin was found forming hydrogen bonds with COX-2 protein, such as the amino acid residues of TYR385, PHE529, LEU531, GLY533, LEU534 with bond distance (BD) of 3.00, 2.77, 3.22, 3.10, and 3.38 in Å, respectively. Interaction with TYR385, PHE529, and GLY533 were classified as strong HB. Monolaurin displayed hydrophobic interactions with COX-2 protein, such as the amino acid residues of VAL349, LEU352, LEU384, TYR385, TRP387, PHE518, PHE518, VAL523, VAL523, ALA527. Hydrogen bonds were mainly formed at the polar head of monolaurin, while hydrophobic interactions were mainly due to the hydrophobic tail of monolaurin. As it was reported [6] that, TYR385 is the most important residue for targeting the COX-2 protein which was found to be interacted with monolaurin through both HB and HP. With these interactions, acetylated side chain is stabilised and effectively prevents hydrophobic groove above Ser-530 from being accessed through the active site channel. By interacting with other residues which synergistically block the hydrophobic channel leading to the active site of cyclooxygenase-2 (COX-2), where arachidonic acid is unable to be fixed, thus, blocking the synthesis of PG and lowering acute inflammation [18]. With the support of the hydrophobic moieties, monolaurin is also well-anchored to the active cleft of the protein. Paracetamol was also found to be interacted with the residue TYR385 through both strong HB (2.96 Å) and HP. However, it has a less significant dock score (− 6.76 kcal/mol) compared to monolaurin (− 7.58 kcal/mol), followed by only having 8 interactions with the protein making it less stable than monolaurin when bound to COX-2.

A few studies also employed in silico COX-2 inhibitory effect using the protein 5F1A [7, 18–20]. *α*-Linolenic acid (6.833 kcal/mol) was the best docking compound of a reported study which created two strong HB with the amino residue, namely, GLN454 and HIS214 [18]. Carboxyl group of a-linolenic acid interacts with TRP388, LEU390, and TYR385 via three Pi-alkyl type interactions. Another study reported aspirin and its best derivative with binding score of -6.5 kcal/mol and − 7.2 kcal/mol, respectively [19]. The best derivative was found to form carbon HB via HIS207 and HIS386, also, normal HB via THR206 and TYR385. Carbon HBs are formed between normal aspirin and HIS207, HIS386, and normal HB with TYR385. As a validation of the current study, the aspirin docked also formed HB with TYR385, so the prior findings are congruent with our results. Likewise, monolaurin shown the better potential to inhibit COX-2 than the reported compounds in terms of better binding affinity (− 7.58 kcal/mol) and strong HB (TYR385, PHE529, and GLY533) formed via the docking. In another study, four strong HB were formed between a potential drug and ASN382 and THR212 amino acid residues in the active cavity of COX-2 (5F1A) protein as the best docking result feasible for this target [7]. However, the hits where not relevant with important active site main residue which only allows the function of blocking the hydrophobic groove of the protein’s active cavity without inhibiting its function totally. Similarly, a study done by Beura et al. (2020), whereby the docked compound only formed hits with supplementary residues, such as GLN289, THR212, ASN382 and HIS207 using the same protein (5F1A) [20].

Further studies revealed in Table 2 showed that less hydrophobic interactions were exhibited by reference drugs compared to MCMs and MCFAs due to less flexibility of reference drugs present with aromatic group. The carboxyl moiety that provides HP, NI and HA pharmacophore features and interactions is a good inhibitor of COX-2 activity based on structure-based drug design [5]. On the other hand, despite salicylic acid and aspirin having carboxylate moiety does not participate in the binding of residues in active site which provides the explanation for the affinity of binding to COX-2 is not strong [6]. All the MCFAs have carboxyl moiety on the hydrophilic fraction making then suitable for binding to the active pocket of COX-2 protein. Thus, capric acid was able to form salt bridge with COX-2 protein involving the amino acid residues of HIS90. However, none of the reference drugs and other MCFAs have salt bridge interaction with any amino acid residues of the COX-2 protein despite having pharmacophore for NI. Nevertheless, monolaurin still outshined capric acid in terms of binding score and number of interactions throughout the docking.

Upon scrutinizing all the interaction and docking data, pool of facts from the bioinformation collected suggested that monolaurin of VCO may have the greatest in silico inhibitory action against COX-2 to halt the inflammatory responses compared to other reference drugs, MCMs and MCFAs. Then, MDS was applied upon the Monolaurin–COX-2 complex to verify the pharmacophore model descriptors, verify the poses and binding energies, and study the stability of the interactions of monolaurin within the catalytic region of the COX-2. The Monolaurin–COX-2 complex showing best hydrogen bond and RMSD result throughout 1000 frames of simulation in the MDS study. The hydrogen acceptor (HA) between monolaurin with the side of residue TYR385 of COX-2 protein appears to be long-living throughout the simulation with an occupancy of 67.03%. As mentioned by Lucido et al. (2016), TYR385 is the most important residue for targeting the COX-2 protein which was found to form long-living HB and with a high percentage of occupancy by monolaurin which further determine its stable inhibitory properties [6]. Paracetamol was also a HA to the side of residue TYR385 of COX-2 protein with a high occupancy of 54.85% but was outclassed by the 67.03% occupancy with monolaurin. A MDS research was reported for aspirin, ibuprofen, naproxen, celecoxib and tyrosol ligand complexes of COX-2 (5F1A) [21]. After MDS, the amino acid residues LEU384, TYR385, TRP387, and SER530 stood out as particularly promising for the catalytic activity of this receptor with their high interaction fraction values (0.9, 0.8, 0.95 and 0.8), as well as their participation in the formation of HB with ligand. The results were in accordance with good indication and validation of TYR385 as the main residue of COX-2 protein even upon MDS. In another study the residues that stood out were THR206, THR212, and ASN382 which formed protein–ligand HB and water bridge followed by ligand–protein via the most potent compound’s substituted amine group, amide group and hydroxyl group forming strong HB with THR206 (62%), THR212 (67%), and ASN382 (47%), respectively [20].

Moreover, the Monolaurin–COX-2 complex obtained the best RMSD score of 1.137 $\pm$ 0.153 Å falling in the range of 0.480–1.520 Å throughout 1000 frames of simulation. This indicated the strong binding between the complex and great potential of monolaurin to stably inhibit COX-2 protein. The stability of the ligand within the active site of the receptor can also be represented through fluctuations in the RMSD plot of the ligand [21]. The plot for Monolaurin–COX-2 complex exhibited slight fluctuations compared to other complexes but not determined as large or extreme fluctuations which imply major movement of the ligand from the receptor pocket during simulation. Yadav et al. (2020) reported data of RMSD fluctuations within the aspirin receptor complex seen in the RMSD plot until 5 ns, then stabilised and converged demonstrating consistent behaviour steadily until 10 ns, and maintained to mildly oscillate until 15 ns [21]. Similar trend of RMSD plot was observed with aspirin–COX-2 complex’s RMSD plot which determined that MDS was carried out on the right track. Likewise, three out of the four test complexes of another study showed significant fluctuations in the protein–ligand interaction after 20 ns of simulation time, whereas the optimal complex displayed fluctuations for just the first 9 ns before slowly approaching equilibrium [20].

As evidenced by the low average RMSD of all complexes (< 2 Å), the relatively moderate fluctuations in the RMSD plot, and the good HB with TYR385 especially the Monolaurin–COX-2 complex, it appears that all small molecules formed stable complexes with COX-2 protein. However, a MDS of 100 ns would be suggested to best validate the results of this in silico investigation, which was conducted using only 10 ns. All these results displayed the potential inhibitory effect of VCO derivatives on COX-2 protein with monolaurin showing better interaction to protein compared to other MCMs, MCFAs, and reference drugs.

On the other hand, the efficient metabolism and activity of a drug candidate depends on its absorption and ready availability throughout the body. The issue of toxicity is also crucial, but it is typically overlooked in favour of the ADMET profiling [47]. Clinical trial failures caused by hazardous side effects are extremely costly and damaging to the medication development phase [46]. Opportunities for accelerating the discovery of new targets and, eventually, compounds with predicted biological activity are presented by the combination of in silico drug-likeness prediction and additional ADMET with Toxicity techniques.

All the screened compounds in this study displayed ADMET values within a compliant range when subjected to in-silico ADMET profiling, which was performed on all docked candidates to verify the physicochemical and pharmacokinetics properties. Through Osiris, MCMs, namely, monolaurin, monocaprin, and monocaprylin, showed no toxicity and low moderate DSC of 41%, 45%, and 47%, respectively, better than reference molecules. The cLogP values for MCFAs ranged in 2.01–6.05, while MCMs ranged in 1.04–5.08. All the tested compounds showed good TPSA values without exceeding 160. MCMs are active enzyme inhibitor (EI) ranging from 0.10 to 0.27 and no RO5 violation which was computed by Molinspiration. MCMs such as monolaurin, monocaprin, monocaproin, monocaprylin, and monomyristin and only MCFAs, namely, capric acid, caprylic acid, lauric acid, and myristic acid, exhibited passing BBB penetration, Caco-2 permeability, HIA, P-glycoprotein inhibitor/substrate, hERG inhibitors, CYP450 inhibitor/substrate, carcinogens, AMES mutagenicity, honeybee toxicity, fish toxicity, tetrahymena pyriformis toxicity, aqueous solubility and rat acute toxicity predicted via AdmetSar.

## Conclusions

A pharmacophore visualization was done based on the active structure of VCO derivatives. Monolaurin exhibited two HD, three HA and five HP pharmacophore features. The in-silico molecular docking, MDS results verified that the Monolaurin–COX-2 complex was the preferred complex. The conclusion was based on their corresponding lowest binding energy of − 7.58 kcal/mol with 15 interactions with the residues in active pocket of COX-2. Monolaurin was found forming hydrogen bonds with COX-2 protein such as the amino acid residues of TYR385, PHE529, LEU531, GLY533, LEU534 and displayed hydrophobic interactions with COX-2 protein, such as the amino acid residues of VAL349, LEU352, LEU384, TYR385, TRP387, PHE518, PHE518, VAL523, VAL523, ALA527. Strong hydrogen bonds were depicted between residues, namely, TYR385 (3.00 Å), PHE529 (2.77 Å), and GLY533 (3.10 Å) and monolaurin in the active cavity of COX-2. RMSD value (1.137 $\pm$ 0.153 Å) and HB (67.03% occupancy of TYR385) investigations of Monolaurin–COX-2 complex through MDS strongly support these findings. Redocking of this complex still maintained a strong hydrogen bond (2.87 Å) with the main residue TYR385. Nevertheless, a MDS of 100 ns would be suggested to best to validate the results of this in silico investigation, which was conducted using only 10 ns. Altogether, our findings revealed that potential inhibitory effect of VCO derivatives on COX-2 protein with monolaurin showing better interaction to the protein residues compared to other MCMs, MCFAs, and reference drugs. As indicated by this in-silico study, monolaurin passed all the ADMET may be the new therapy for relieving inflammation by suppressing the synthesis of prostaglandin via COX-2 protein.

In addition, this study opens futuristic in vitro and in vivo test possibilities with appropriate models for VCO, VCO derivatives and especially monolaurin to further authenticate the anti-inflammation properties.

## Data Availability

The data sets used and/or analysed during the current study are available from the corresponding author on reasonable request.

## Abbreviations

ILE: Isoleucine
KI: Kinase inhibitor
LEU: Leucine
logS: Solubility
M: Mutagenicity
MCFA: Medium chain fatty acid
MCM: Medium chain monoglyceride
MCT: Medium chain triglyceride
MDS: Molecular dynamic simulation
MET: Methionine
NI: Negative ion
NPT: Isothermal–isobaric
NRL: Nuclear receptor ligand
NSAIDs: Nonsteroidal anti-inflammatory medications
PG: Prostaglandin
PGP: P-glycoprotein
PHE: Phenylalanine
PI: Protease Inhibitor
PI: Positive Ion
RMSD: Root mean square deviation
RO5: Rule of five
RT: Reproductive development toxicity
T: Tumorigenicity
TPSA: Molecular polar surface area
TRP: Tryptophan
TYR: Tyrosine
VAL: Valine
VCO: Virgin coconut oil
